# Movement-reactor oven and wire mesh filter for large-scale solvothermal preparation and purification of silver nanowires with high uniformity in length and diameter for the fabrication of low and high haze transparent conductive films

**DOI:** 10.1039/c9na00189a

**Published:** 2019-05-27

**Authors:** Mohammad-Reza Azani, Azin Hassanpour, Nicoló Plaia, Mojtaba Meshkat-Mamalek

**Affiliations:** Department of Research and Development, Institution: Intercomet S.L. Calle Cañada 15, 28860 Paracuellos de Jarama Madrid Spain moha@intercomet.com

## Abstract

Conventional polyol synthesis has been widely used for the preparation of metal nanowires with different aspect ratios. However, its main drawback is that it is difficult to control the reaction parameters for the shape-controlled synthesis of metal nanowires. Although with the solvothermal method the shape control and growth rate improve, but on a large scale, there are still some challenges such as high reactor weight, long processing time and non-heat uniformity in different positions of the oven. In addition, the purification of nanowires on a large scale is another challenge in this area. Herein, we report the use of a movement reactor oven with the ability to rotate the reactors, and high heat conductive, light-weight reactors for the large-scale preparation of silver nanowires with highly uniform lengths and diameters. The uniformity of the length and diameter of the nanowires using the movement reactor oven in comparison with that using the fixed-reactor oven significantly improved. Furthermore, the amount of silver nanowires increased by 20 times using the new reactor in comparison with the standard Teflon-lined stainless steel reactor for the same reaction time. Reactor rotation, as a new parameter to adjust the final product, was introduced. The synthesized nanowires with and without rotation showed the same morphology and electrical conductivity. For the purification step, first, a new angular filtration method using a wire mesh filter was applied to separate the solvents and ionic impurities, and then by using the same filter based on tangential flow, the nanowires were separated from nanoparticles. Finally, uniform and coffee ring-free transparent conductive films (TCFs) with different haze values were fabricated by coating the formulated silver nanowire ink containing a mixture of green solvents. The fabricated films can be potentially used in a wide range of applications.

## Abstract 2

Large scale synthesis and purification of highly uniform silver nanowires to fabricate transparent conductive films are reported.

## Graphical abstract

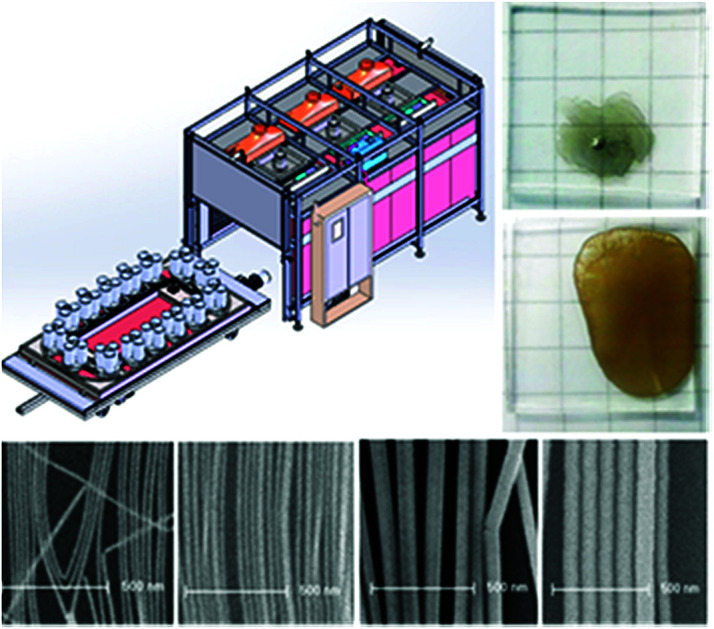

## Introduction

In the last decade, one-dimensional (1D) nanostructures have attracted great attention due to their fascinating physical properties and potential technological applications.^1–5^ Among them, the attention to networks of metal nanowires and nanorings, especially in the case of silver, has been increased for the fabrication of transparent conducting films (TCFs).^6^ TCFs based on silver nanowires have been successfully used in organic solar cells^7^ and light-emitting diodes (LEDs),^8^ highlighting silver nanowires as promising opto-electrical materials with higher performances than other opto-electrical materials (transparent conductive oxide (TCO), graphene, carbon nanotubes and PEDOT:PSS).^9,10^ In fact, in this category, only metallic nanowires have high haze values and transparency in the IR region. These specifications highlight the potential of metallic nanowires for solar cell and LED applications.^11^

Depending on the diameter and length of nanowires, they can be classified for different applications. For example, touch screens or displays need TCFs with low haze and high transparency, which can be produced by using ultra-thin nanowires with a high aspect ratio,^12^ while some applications such as solar cells need high haze TCFs to increase light scattering and trapping light inside the active layer to increase the efficiency of the device. Accordingly, thick nanowires are preferable for the fabrication of high haze TCFs.^13^

To date, several fabrication processes have been reported for the preparation of silver nanowires such as polyol processes, solvothermal methods, photo-reduction techniques, electro-deposition processes, ultraviolet radiation techniques and DNA template methods.^14^ Among them, the solvothermal method is the most appropriate to produce shape-controlled nanowires and potentially can be used for large scale production. However, the challenges associated with the large scale production of nanowires *via* solvothermal methods are high, including reactor weight, long processing time and unconventional heat in different positions of the oven. To obtain uniform nanostructure materials in different reactors, receiving uniform and same heat by reactors is critical. Solvothermal methods normally lead to products consisting of a mixture of nanowires and nanoparticles. The presence of nanoparticles has an adverse effect on the optical and/or electrical properties of the final product. A higher ratio of nanoparticles/nanowires can be obtained during the synthesis of thinner nanowires, which are generally preferred to make low haze TCFs.^15^ Thus, an additional purification step is usually needed in this method to remove the nanoparticles present in the final products.

To date, different purification techniques such as dead-end filtration, centrifugation, gel electrophoresis, dialysis, selective precipitation and cross-flow filtration have been applied to purify nanowires. However, the reported methods have several limitations. For example, simple dead-end filtration can add impurities to the filter cake and damage the nanowires. Also, nanowires can suffer deformation and aggregation under centrifugation.^16^ Gel electrophoresis and dialysis as highly effective purification methods are time-consuming processes and difficult to scale up for large scale production.^17^ The use of selective precipitation agents for the purification of metal nanostructures is another reported method. Specifically, a solvent such as acetone is used to aggregate and precipitate metal nanowires and separate them from nanoparticles and other impurities.^10^ However, the use of large amounts of non-environmentally friendly solvents is difficult to apply for large scale purification. A tangential flow (cross-flow) filtration method was used for the large-scale purification of a nanowire suspension to remove nanoparticles and other impurities.^18^ However, the filters commercially available for cross-flow filtration have small pore sizes, which lead to enhanced aggregation of the nanowires. In addition, their high price, difficult cleaning and few-time use are other drawbacks.

Herein, we report the large-scale preparation of uniform silver nanowires with controllable diameters using a home-designed and home-made rotational oven and high heat conductive light-weight reactors. Also, for the first time, the effect of reactor rotation on the final product was investigated. For the purification step, a highly efficient and environmentally friendly method using home-designed and home-made stainless steel filter based on the tangential flow (cross-flow) filtration method was used. In the purification process, first, a new angular filtration method was applied to separate the solvents and ionic impurities from the nanowires and particles, and then using a home-designed and home-made cheap and high-life filter based on tangential flow, the nanowires were separated from the nanoparticles. TCFs with different optical properties were fabricated by coating silver nanowire ink using an adjustable coater on PET substrates.

## Results and discussion

### (a) Preparation of silver nanowires using three different ovens

To determine the effect of heat uniformity on the final product, three different ovens, a natural convection oven, a forced air circulation oven without reactor rotation and a forced air circulation oven with the ability to rotate the reactors (movement-reactor oven), were selected.

**Scheme 1:**
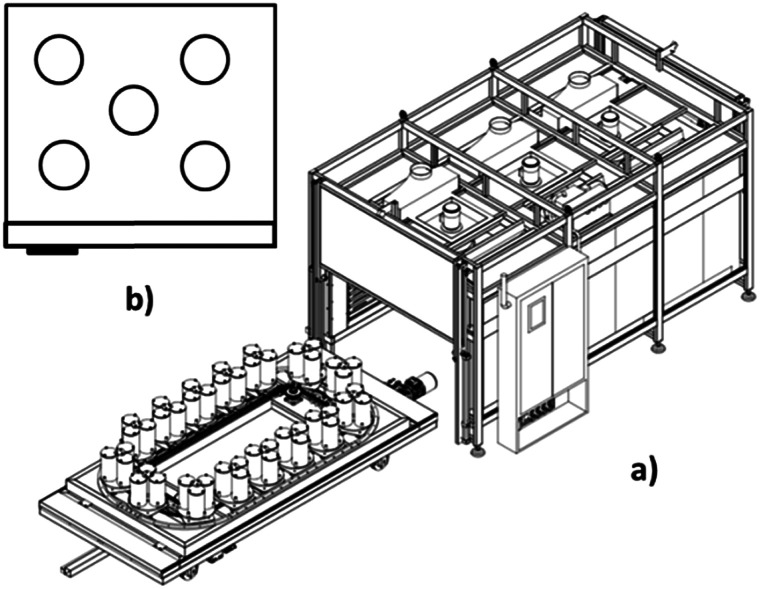
(a) Schematic of designed and fabricated forced air circulation movement-reactor oven for the production of metal nanowires. (b) Top view of the reactor positions in a natural convection oven and forced air circulation oven.

Scheme 1a shows the designed and fabricated forced air circulation movement reactor oven for the production of metal nanowires, which contains two main parts. One part is the circular line, which holds several platforms with the capacity of at least 1 reactor, and the second part is the heating instruments.

The mentioned ovens were used for the reactors (5 units) containing the same reaction mixture (to produce nanowires) in different positions of each oven (Scheme 1b). The range of diameter, length and yield of the synthesized silver nanowires in different positions for each oven are shown in Table 1. The nanowire yields were calculated based on our recent publication.^19^

**Table 1:** Characterization of the silver nanowires obtained in the different ovens

Oven	Diameter (nm)	Length (μm)	Yield (%)
Fixed reactor natural convection	17–45	10–45	0–52
Fixed reactor forced air circulation	18–32	15–40	20–51
Movement-reactor forced air circulation	17–22	15–25	46–52

It is clear that the nanowires obtained using the movement-reactor oven (with translational movement of 1 rpm) have the most uniform diameters and lengths and greatest yields in all the samples obtained from the five reactors. However, the products obtained from the natural convection oven and forced air circulation oven were different among the 5 reactors placed in different positions in these ovens. Therefore, the diameter and length range for these products was broader than that for the products obtained in the movement-reactor oven. In the natural convection oven, there was 3% error in temperature uniformity, while in the forced air circulation oven it was lower (1.33%). Thus, at 150 °C, the temperature uniformity was 4.5 °C and 2 °C for the natural convection and air circulation ovens, respectively.

It is interesting that in the natural convection oven, after the reaction was completed, there was a reactor with only nanoparticles (0% yield of nanowires). This observation confirms the presence of a very high temperature-sensitive reaction for ultra-thin nanowires. It is worth mentioning that this sensitivity decreases with an increase in the diameter of the nanowires. For example, in the synthesis of nanowires with a higher diameter (*D* > 70 nm), small changes (around 10%) in the yields of the reactors in the natural convection oven were observed. This observation can be related to the nanowire diameter range. In the case of ultra-thin nanowires, the range of diameter thickness is ±3, but in the case of thick nanowires (70 nm), this range is ±15. This means that the category of ultra-thin nanowires is limited by 5 times in comparison with nanowires with a diameter of 70 nm. Thus, by reducing this range, the sensitivity of the reactions will increase.

According to the results, the uniformity in the final product is higher in the forced air circulation oven due to the lower error in temperature. This uniformity in the final product was improved by rotating the reactors inside the oven. By rotating the reactors inside the oven, all the reactors received the same amount of heat, and thus the final products in all the reactors were the same.

### (b) Synthesis of silver nanowires in different reactors

To date, the standard solvothermal reactors are Teflon-lined stainless steel reactors, which are available in different sizes. The drawbacks of standard reactors, specifically for the large-scale synthesis of nanomaterials, are their heavy weight due to their big size and the presence of stainless steel as a weak heat conductive material, which lead to an increased reaction time. In addition, the presence of a thick-wall Teflon vessel inside the reactor can also increase the reaction time. Herein, we designed and fabricated new light and high heat conductive reactors to prepare uniform silver nanowires in a shorter time. These reactors were made of aluminum coated with a very thin layer of Teflon. Table 2 shows the reactor conditions and specifications of the prepared silver nanowires from one reaction in different reactors.

**Table 2:** Characterization of the silver nanowires obtained in different reactors

Reactor	Filled vol. (mL)	Temp. (°C) time (h)	Yield (%)	Nanowires *D* (nm) *L* (um)
Teflon-lined S.S (50 ml)	35	160 °C – 7 h	91	70 (20–50)
Teflon-lined S.S (1 L)	700	160 °C – 7 h	0	Only nanoparticles
Teflon-coated Al (1 L)	700	160 °C – 7 h	70	40 (20–35)
Teflon-coated Al (1 L)	700	Temp. prog. – 7 h	89	70 (20–50)

It is clear that by increasing the Teflon-lined stainless steel reactor size from 50 mL to 1000 mL (20 times), there was no nanowires at the same temperature and the yield of the nanowires reached zero from more than 90%, but by using the Teflon-coated aluminum (1 L) reactor under the same reaction conditions, the yield of the nanowires increased to 70%.

It is interesting that by adjusting and controlling the temperature gradient in four steps (from room temperature to 180 °C in 30 min, at 180 °C for 60 min, from 180 °C to 160 °C in 30 min, and at 160 °C for 5 h) in the designed aluminum Teflon-coated reactor, it was possible to obtain 20 times more silver nanowires at the same reaction time (7 h) with the same range of length, diameter and yield of nanowires compared to that with the 50 mL Teflon-lined stainless steel reactor. In fact, in the new reactors, by changing the stainless steel with a high heat conductive material (Al) and by reducing the thickness of the Teflon vessels from a few millimeters to less than 200 μm as a coating inside the reactor, the heat conductance of the reactor extremely increased and the reaction time decreased.

The results in Table 2 also confirm the role of the temperature-gradient in the final products. We successfully adjusted the gradient of reaction temperature in the 1 L Teflon-coated aluminum reactor to obtain the same product as that in the 50 mL Teflon-lined stainless steel reactor.

### (c) Synthesis of nanowires with different diameters in a circular oven

By selecting the movement-reactor forced air circulation oven and a big and light-weight Teflon-coated aluminum reactor, the parameters of the reaction to produce silver nanowires with different thickness for different applications were optimized. Herein, by using some additive salts under different conditions (Table 3), uniform silver nanowires were obtained on a large scale. As shown in Table 3, silver nanowires with a uniform thickness of 17 nm, 35 nm, 70 nm and 100 nm were synthesized.

**Table 3:** Additive salts, temperature and the specification of synthesized silver nanowires in large Teflon-coated aluminum reactors

Number	Additive salts	Temp. – time	Diameter (nm)	Length (μm)
Reaction 1	TPA-C, KBr	Temp. program – 7 h	17 ± 3	15–25
Reaction 2	TPA-C, KBr	Temp. program – 7 h	35 ± 10	30–50
Reaction 3	NaCl	Temp. program – 7 h	70 ± 15	40–70
Reaction 4	BMIM-Cl	Temp. program – 7 h	100 ± 20	50–90

The mechanism and selection of different salts for the solvothermal synthesis of silver nanowires were reported in our previous work.^19^ Generally, in this synthesis, the effect of the anion in the salt on the diameter of the nanowires is more significant than that of the cation. Normally, silver nanowires with diameters larger than 70 nm can be obtained easily in high yield only in the presence of salts with chloride (for example, herein, NaCl for diameters of 70 nm and BMIM-Cl for diameters of 100 nm were used). To synthesize high-yield thin diameter nanowires (less than 40 nm), a mixture of chloride and bromide ions was used, as recently reported.^20,21^ In this mixture, by decreasing the chloride/bromide ratio, the diameters of the nanowires will be reduced. In addition to the synthesis of thin nanowires, the positive effect of the presence of pressure during the reaction and the use of an ionic liquid as a soft template were reported.^22^ Herein, by using the ionic liquid, TPA-C, and KBr as the source of chloride and bromide ions, respectively, in the presence of pressure (150 kPa), thin (35 nm) and ultra-thin (17 nm) nanowires were synthesized. The ratio of TPA-C/KBr for silver nanowires with a diameter of 35 and 17 nm was 3.0 and 1.61, respectively.

**Fig. 1:**
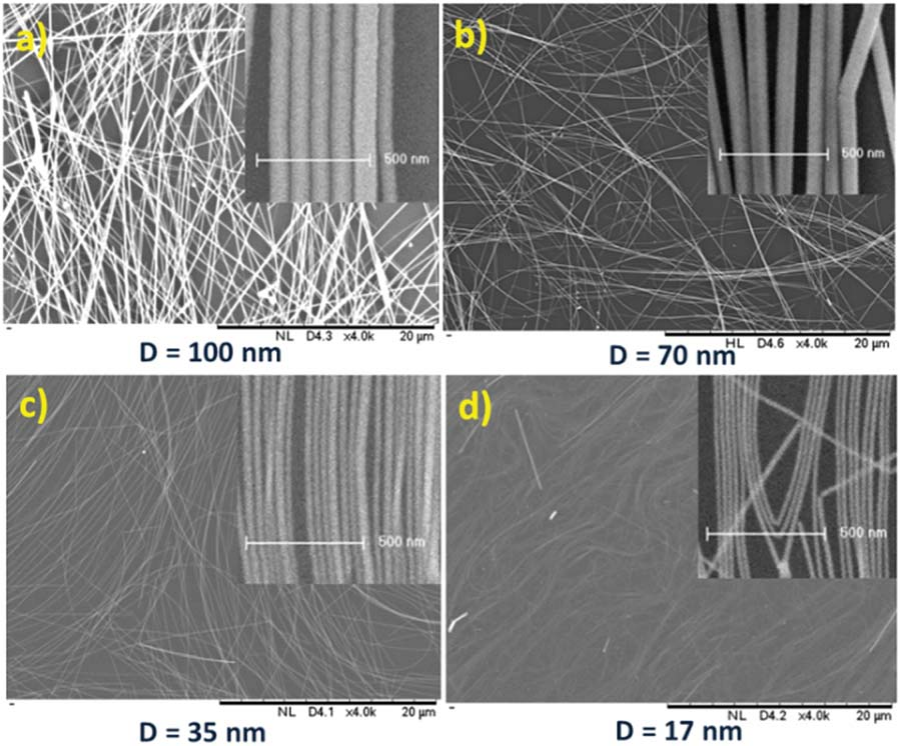
SEM images in the same order of magnitude of silver nanowires with uniform diameter thickness with an average of (a) 100 nm, (b) 70 nm, (c) 35 nm and (d) 17 nm on a glass substrate by drop casting (inset of each image: HR-SEM in the same order of magnitude to measure the thickness).

The SEM (insets: high-resolution SEM to measure and check the diameter and uniformity) images of the synthesized and purified nanowires are shown in Fig. 1, which confirmed the presence of nanowires with uniform diameter free of nanoparticles.

**Fig. 2:**
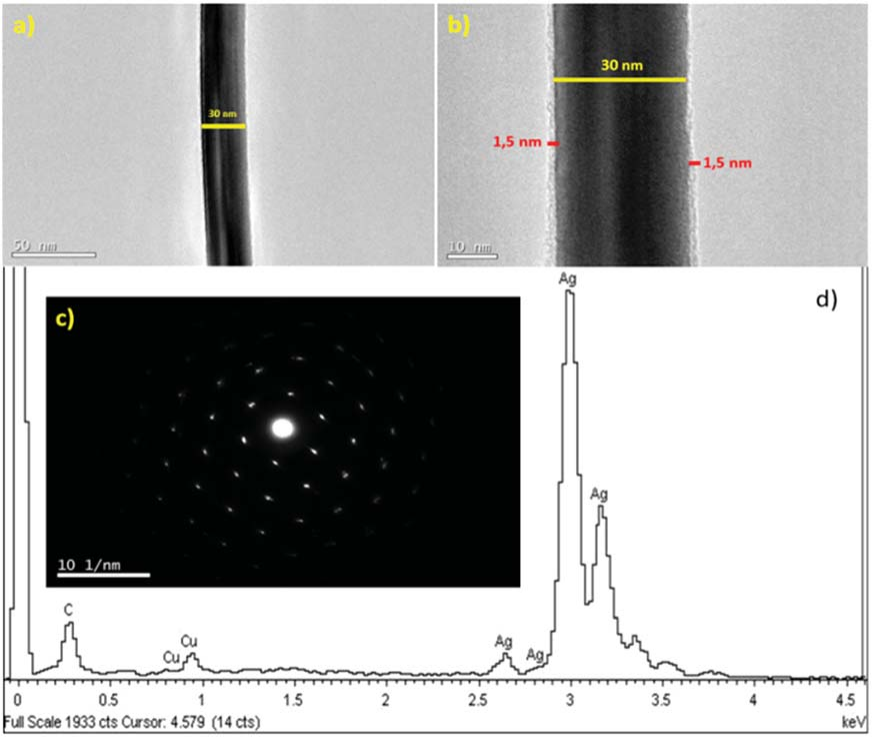
(a) TEM image of an individual silver nanowire. (b) High-resolution TEM image of PVP layer (about 1.5 nm thickness) on silver nanowire. (c) Electron diffraction pattern of a randomly selected silver nanowire. (d) EDX spectrum of the silver nanowire.

Fig. 2a presents the TEM image of a silver nanowire. It can be seen that the nanowire has a uniform diameter. In the higher magnification image of the silver nanowire, as shown in Fig. 2b, a PVP layer with a thickness of about 1.5 nm was covered on the nanowire surface and the border of two panels in the polygonal nanowire structure is observed. The selected area electron diffraction (SAED) pattern along the 〈011〉 zone axis in Fig. 2c further exhibits the single crystalline nature of the silver nanowires, which is consistent with the observations in the literature.^23^

Fig. 2d presents the EDX spectrum of the silver nanowire, which shows that the nanowires are composed of pure silver and the possibility of salt elements in the samples is excluded.

### (d) Effect of centrifugal rotation of reactors

The fabricated movement-reactor oven not only can rotate the reactors with translational movement, but also can rotate the reactors with centrifugal rotation at different speeds. In this part, the effect of centrifugal rotation of the reactors at different speeds was investigated. Table 4 shows the effect of rotating the reactors around themselves on the silver nanowire specifications (diameter, length and yield) by increasing the centrifugal speed. At low speed, there was no change in diameter, while the length and yield increased. Thus, by increasing the length with the same diameter, the aspect ratio (length (nm)/diameter (nm)) increased. Having high aspect ratio nanowires is preferable in some applications such as the fabrication of TCF for displays. Another advantage of low centrifugal speed is that it results in a higher uniformity in length (smaller length range). At a high centrifugal speed, the diameters of the nanowires also increased. As already published for the polyol method, by increasing the magnetic stirring speed (in the range of 300 to 1000 rpm), the possibility of Ag atoms and multi twin particles coming together is reduced and the formation of thinner and shorter nanowires increases.^24^ In contrast, herein, by the solvothermal method, the very slow reactor movements increased the motion of particles. Thus, the possibility of joining Ag atoms and multi twin particles to produce thicker and longer nanowires than a stagnant reactor increased. According to these results, the rotation of the reactors during the reaction can affect the final product. Thus, herein, for the first time, centrifugal rotation is reported as an important parameter to obtain optimized silver nanowires.

**Table 4:** Effect of centrifugal speed on the specifications of the nanowires

Speed (rpm)	Diameter (nm)	Length	Yield (%)	Aspect ratio	Morphology	*σ* × 10^5^ (S m^−1^)
0	60	20–50	75	583	Pentagonal	1.7
10	60	55–70	90	1042	Pentagonal	2.38
50	150	70–120	95	633	Pentagonal	4.5

**Fig. 3:**
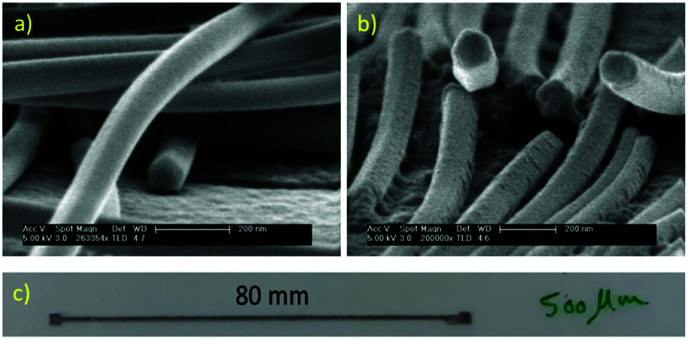
HR-SEM images of the synthesized silver nanowires with (a) 10 rpm and (b) 50 rpm. (c) Printed line of synthesized silver nanowires with 10 rpm rotation speed.

To check the morphologies of the synthesized nanowires with different rotation speeds (10 and 50 rpm), high-resolution SEM images were taken, as shown in Fig. 3a and b, respectively. It can be seen that the nanowires have the same pentagonal structures as that in all the previous reports on the solvothermal synthesis of silver nanowires.^19^ A comparison of the electrical conductivity of synthesized nanowires with and without rotation was not possible due to the big difference in their aspect ratio. We printed some lines on a PET substrate and obtained the electrical conductivity (Table 4). To compare the electrical conductivity, two groups of nanowires with a close aspect ratio were selected: nanowires synthesized using 10 rpm rotation speed (*D*: 60 nm, *L*: 55–70, and aspect ratio 1042) and nanowires synthesized without rotation (*D*: 70 nm, *L*: 50–80, and aspect ratio 1071). Two lines with a length of 80 mm, width of 500 μm and height of 5 μm were printed on the PET substrate (Fig. 3c). After measuring the electrical resistances, close electrical conductivity values of 2.38 × 10^5^ (S m^−1^) and 3.5 × 10^5^ (S m^−1^) were obtained for the nanowires with and without rotation, respectively. The small difference can be related to higher diameter thickness and longer nanowires in the case of the nanowires synthesized without rotation. Thus, these results confirm that there was no big change in the conductivity of the synthesized nanowires with and without rotation.

### (e) Purification of silver nanowires by continuous washing system

**Fig. 4:**
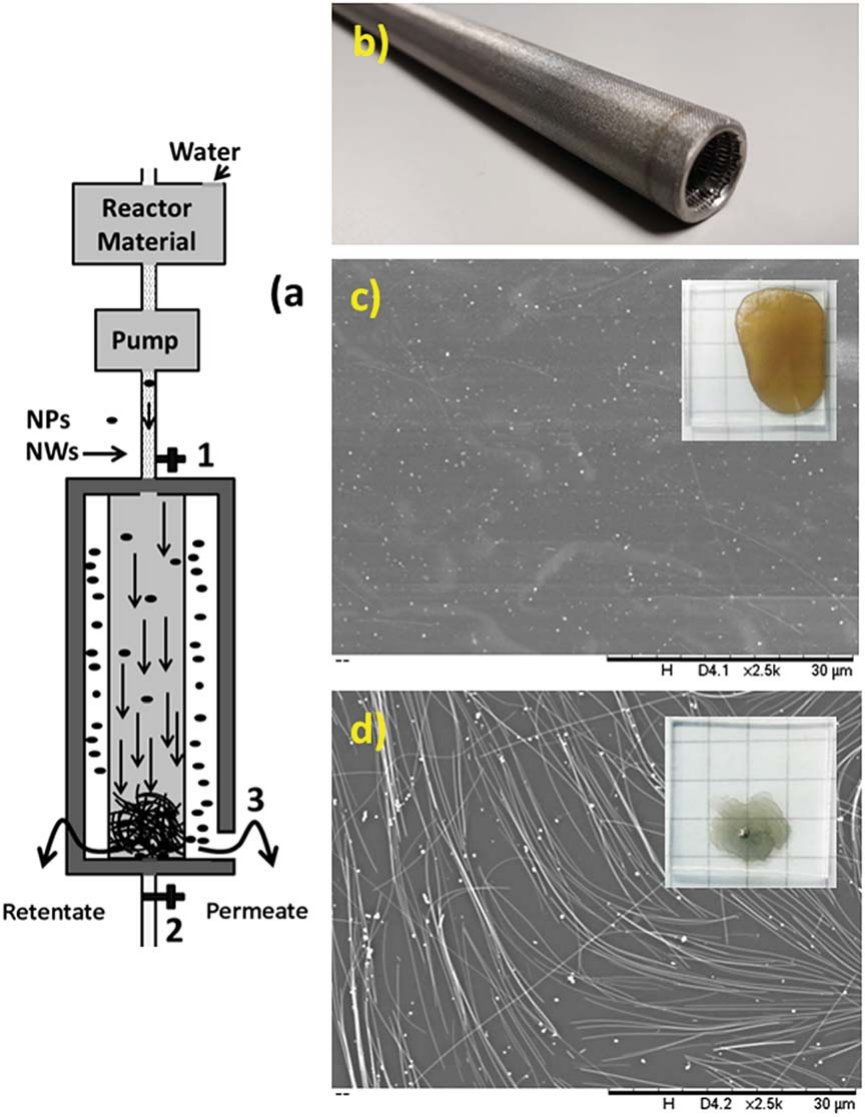
(a) Designed and fabricated stainless steel wire mesh filter for the purification of metal nanowires. (b) Stainless steel wire mesh cylindrical filter with a pore diameter of 2 μm. (c) SEM image of permeate from the filtration (inset: image of dry permeate on a glass substrate). (d) SEM image of retentate from the filtration (inset: image of dry retentate on a glass substrate).

The synthesized uniform nanowires were purified with a home-designed stainless steel wire mesh filter. Fig. 4a shows a schematic of the filtration unit. This filtration unit comprised a filter housing with an inlet (1) and outlet (2) and a second outlet (3) (Fig. 4a). Inside the filtration unit, there was one stainless steel wire mesh cylindrical filter with a pore diameter of 2 μm (Fig. 4b). This filter was located within the housing. Thus, the filtration unit allowed fluid communication between the inlet (1) and the two outlets.

The reaction mixture contained ethylene glycol, nanowires, nanoparticles, unreacted salts, ions and unreacted polymer. Herein, in the first step, most of the ethylene glycol, unreacted salts, ions and unreacted polymer were removed by “angular filtration”, as shown in Fig. 4a (angular filtration refers to a filtration process wherein the feed-flow and the filtration-flow direction form an angle between 90° and 180°). During the angular filtration step, the outlet (2) of the filtration unit was closed. Therefore, the fluid circulated from the inlet (1) to the outlet (3), passing through the stainless-steel filter.

After this step was completed, most of the ethylene glycol, unreacted salts, ions and unreacted polymer and some nanoparticles were extracted as permeates. The SEM image of the permeate in Fig. 4c shows the presence of nanoparticles free of nanowires as conductive materials.

This result confirms the functionality of the stainless steel wire mesh filter with a 2 μm pore size for this separation. The inset in Fig. 4c shows the permeate after the evaporation of ethylene glycol. The brownish color and non-conductive residue on the glass substrate confirm the presence of salts, ions and unreacted polymer together with nanoparticles. In the retentate, almost all the nanowires and some particles, salts and ethylene glycol formed a filter cake. Then, to remove the excess ethylene glycol and salts, after passing the entire reaction mixture through the filter, deionized water was passed through the filter to wash the filter cake (or retentate). The SEM image of the retentate in Fig. 4d shows the presence of silver nanowires with some nanoparticles as conductive materials. The inset in Fig. 4d shows the retentate after the evaporation of water. The grey color and very high conductivity confirm the presence of conductive materials (nanowires and nanoparticles) free of ethylene glycol, unreacted salts, ions and unreacted polymers.

In the second step, by opening valve 2 and closing valve 3, the retentate dispersed and diluted in deionized water to form a feed-flow comprised of nanowires and nanoparticles. The dilution factor has a direct relation with the amount of nanoparticles. As shown in Table 5, in the purification of the nanowires with a thinner diameter, the amount of nanoparticles is higher, and thus the dilution factor for more efficient separation and time for separation should be higher.

**Table 5:** Optimized tangential filtration parameters and percentage of nanoparticles inside the nanowire dispersion before and after purification

AgNW diameter	Dilution factor	Washing time (min)	Flow rate (mL min^−1^)	AgNPs (%) (before–after filtration))
20	100	180	65	60–5
40	40	120	130	40–2
70	20	60	260	20–0

**Fig. 5:**
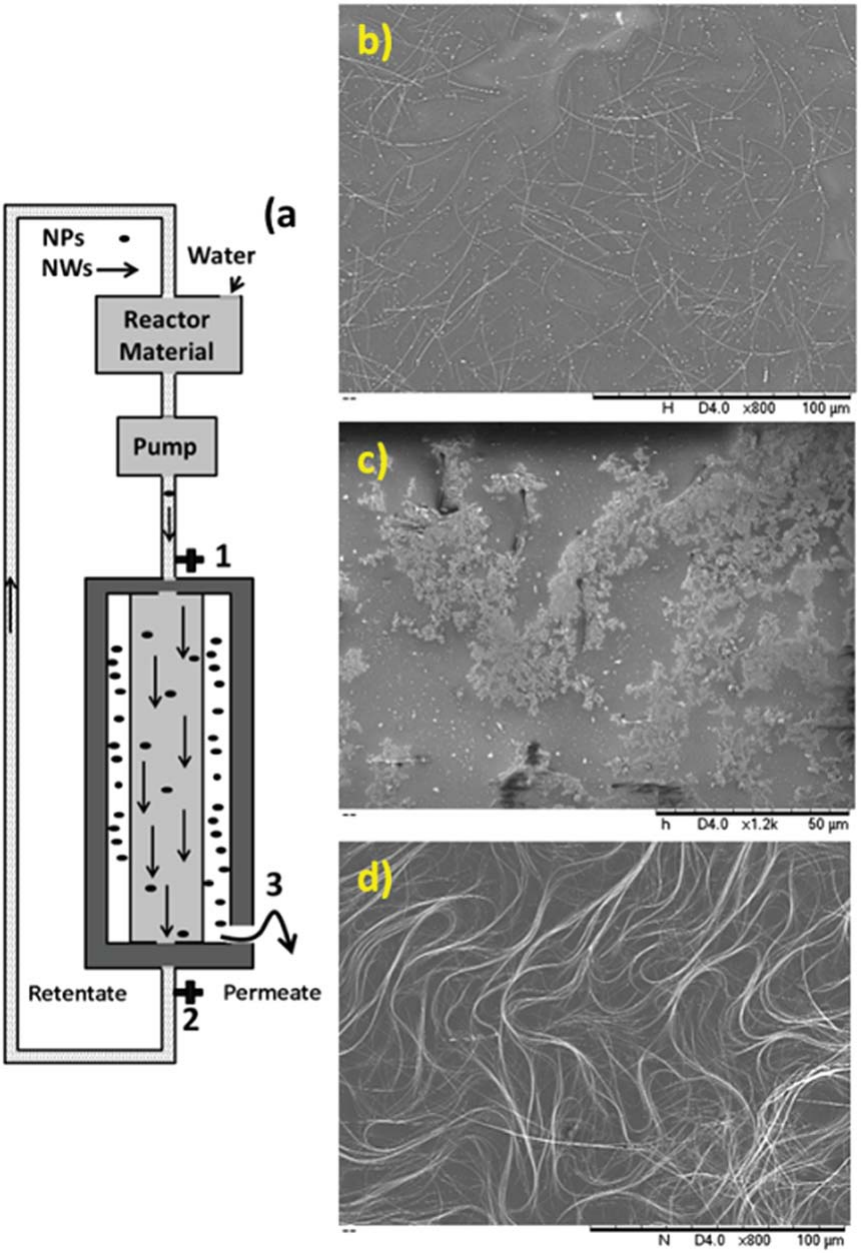
(a) Tangential filtration step of stainless steel filter to separate nanowires from nanoparticles. (b) SEM image of a mixture of nanowires (*D*: 70 nm) and nanoparticles in water before tangential filtration. (c and d) SEM images of permeate and retentate after filtration, respectively.

Then, as shown in Fig. 5a, the tangential filtration step was performed by opening outlet 3. As an example for silver nanowires with a diameter of 70 nm, the diluted feed-flow (20 times) was recirculated through the filter at a flow rate of 260 mL min^−1^ for 60 min to separate the nanoparticles from the nanowires. In this step, the nanoparticles present in the feed flow were selectively separated as permeate from the nanowires (as retentate) by traveling tangentially across the surface of the filter while being recirculated. Fig. 5b shows the mixture of nanowires and nanoparticles in water before cross-flow filtration. Fig. 5c and d show the SEM images of the permeate and retentate, confirming the efficient separation of the nanoparticles from nanowires after 60 min.

In the last step, outlet (2) was closed again and all the excess deionized water was extracted through the filter from outlet (3). Then, the purified filter cake or retentate inside the filter comprising only nanowires was dispersed in different solvents for further characterization and storage. Table 5 shows the percentage of silver nanoparticles in the solids obtained by optimizing the different parameters in the purification process. It is worth noting that these results can be improved by increasing the time or/and increasing the dilution factor of the purification process. This purification method can be easily extended on a large scale by using long multi-wire mesh filters inside one filter housing.

### (f) Fabrication of transparent conductive film

**Fig. 6:**
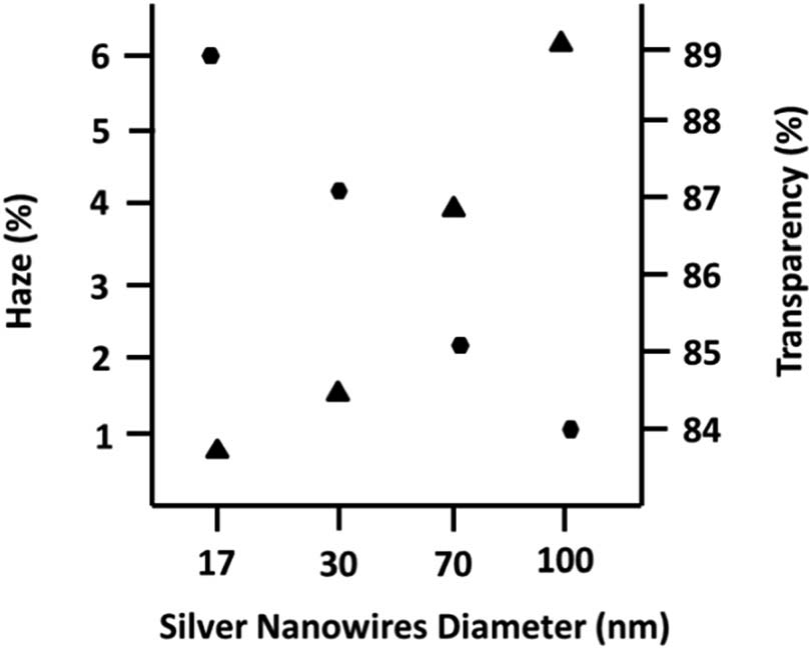
Optical properties of the fabricated film using silver nanowires with different diameters. Circles and triangles are related to the transparency and haze values, respectively.

To monitor the opto-electrical properties of the obtained nanowires, they were dispersed in a mixture of solvents and then coated on PET substrates using an adjustable coater. This mixture containing different solvents (water : ethanol : isopropanol : ethylene glycol) with various viscosities and boiling points permitted us to make conductive ink with good wettability and high adhesion to the PET substrate. The fabricated films exhibited uniform conductivity throughout the entire film. Fig. 6 shows the relation between the diameter of the nanowires and their optical properties (haze and transparency). Although, the length of the nanowires as another important parameter plays a key role in the optical properties of the film, herein, the close aspect ratios of the synthesized nanowires permitted us to compare and determine the effect of diameter on the opto-electrical properties of their films. As shown in Fig. 6, it is clear from the same sheet resistance in all the films (60 ohm. per sq.) that the opto-electrical parameters (lower haze and higher transparency) in the film with a thinner diameter improved compared to the films with a thicker diameter. In fact, low haze (*T*% = 89 and *H*% = 0.8) and high haze (*T*% = 84 and *H*% = 6.2) TCFs were fabricated using silver nanowires with an average diameter of 17 nm and 100 nm, respectively. Thus, we fabricated TCFs with different haze values by having reasonable opto-electrical properties, which can potentially result in a wide range of silver nanowire applications.

## Conclusions

In this study, the large scale preparation and purification of uniform metal nanowires (silver nanowires) with controllable diameters were reported. For their preparation, a home-designed and home-made air circulation oven with the ability to rotate the reactors and high heat conductive, light-weight reactors (Teflon-coated aluminium) were used. This oven can also be used to prepare other chemicals and nanomaterials if very high temperature-sensitive reactions are required, such as the preparation of zero-valent transition metal nanowires. For the first time, the effect of the centrifugal rotation of the reactors on the final nanowire shape was investigated. Long and thick nanowires in higher yield were obtained using a higher centrifugal speed. Also, the reactor rotation, as a new parameter to adjust the final product, was introduced. The synthesised nanowires with rotation showed the same morphology and electrical conductivity. For the purification step, first, a new angular filtration method with a home-designed and home-made stainless steel unit having a 2 μm pore size filter was applied to separate solvents and ionic impurities from the nanowires and particles, and then by using the same filter, based on tangential-flow and optimizing the flow rate and time, the nanoparticles were separated from the nanowires. This purification method can be expanded for other nanowires, nanorods and nanotubes. Finally, TCFs were fabricated on PET substrates with different haze values by dispersing the nanowires in a mixture of low boiling point solvents.

## Experimental

In this study, the following ovens were used:

(a) *Convection oven*: where thermal energy is transferred by convection and radiation to the chamber load. The convection oven used was a NahitaTM series 631 PLUS.

(b) *Air circulation oven*: where thermal energy is transferred by convection and radiation to the chamber load, and a fan accelerates the heat transfer (convection) and air exchange and distributes the temperature homogeneously within the chamber. The air circulation oven used was a POL-EKO TM model SLW 400.

(c) *Movement-reactor oven*: comprises of a conveyor with platforms. The platforms are able to carry 1 to 5 reactors depending on the dimensions of the reactors and the platform. Reactors can be rotated on their axis as centrifugal rotation or/and circulated on the conveyor. Thermal energy is transferred by convection and radiation to the thermally insulated chamber and a fan accelerates the heat transfer (convection) and air exchange and distributes the temperature homogeneously within the chamber.

Polyvinyl pyrrolidone (PVP) powder in ethylene glycol (EG) solution was added to a 50 mL round bottom flask. The mixture was then heated to 110 °C in an oil bath with stirring until the temperature stabilized for 2 h. Then the reaction mixture was left to cool to room temperature. Then, the PVP solution (8.83 mg mL^−1^) was transferred to a 50 mL solvothermal reactor and salt(s) (mole ratio for 17 nm : 1.61 of tetra butyl ammonium chloride (TPA-C)/potassium bromide (KBr), 35 nm : 3.0 of TPA-C/KBr, 70 nm : 7.8 μmol of sodium chloride (NaCl), and 100 nm : 2.9 μmol 1-butyl-3-methylimidazolium chloride (BMIM)) were added quickly to the PVP solution. Subsequently, silver nitrate (AgNO_3_) solution (10.71 mg mL^−1^) was added quickly to the mixture and stirred for 5 min. Then the reactors were placed in a pre-heated oven at 130–180 °C with temperature programming (depending on diameters) for 7 h. After cooling, purification was conducted using a stainless steel wire mesh based on an angular and cross flow filtration. Finally, the obtained silver nanowires were re-dispersed in deionized water by mild stirring for characterization. Conductive inks to fabricate TCFs were prepared by dispersing the synthesized nanowires in a mixture of green and low boiling solvents. Ethanol : water : iso-propanol : ethylene glycol in a ratio of 77 : 11 : 6 : 6 was used as the ink medium. Coating was performed using an adjustable coater (60 μm) on PET substrate. After coating, the samples were dried on a heater at 80 °C for 2 min.

### Printing conductive line

First, using a laser, rectangular spaces with a length of 80 mm and width of 500 μm by cutting double-sided adhesive plastic (SKC) were created. Then these films were fixed on PET substrate and with good control, the dispersion of nanowires in ethanol (10 mg mL^−1^) was inserted into the gaps and the samples were dried at 100 °C for 5 min. After drying, the adhesive tape was removed, and the thickness of the line was measured using a digital micrometer. Electrical resistance was measured using an Amprobe 5XP-A Compact Digital multi-meter.

SEM images were obtained *via* FE-SEM on a PhilipsXL30 S-FEG. TEM images were obtained on a JEOL JEM 2100 transmission electron microscope at an accelerating voltage of 200 kV. All TEM samples were prepared by drop casting dispersions on carbon-coated copper grids.

## Conflicts of interest

There are no conflicts to declare.

## Supplementary Material

## References

[cit1] Liu S., Tang Z. R., Sun Y., Colmenares J. C., Xu Y. J. (2015). Chem. Soc. Rev..

[cit2] Weng B., Liu S., Tang Z. R., Xu Y. J. (2014). RSC Adv..

[cit3] Liu S., Weng B., Tang Z. R., Xu Y. J. (2015). Nanoscale.

[cit4] Liu S., Han C., Tang Z. R., Xu Y. J. (2016). Mater. Horiz..

[cit5] Tang Z. R., Han B., Han C., Xu Y. J. (2017). J. Mater. Chem. A.

[cit6] (b) Azani M. R. and Hassanpour A., EP 3281723 A1, 2018

[cit7] Basarir F., Sayarirani F., Kosemen A., Camic B. T., Oytun F., Tunaboylu B., Shin H. J., Nam K. Y., Choi H. (2017). Mater. Today Chem..

[cit8] Cheong H. G., Triambulo R. E., Lee G. H., Yi I. S., Park J. W. (2014). ACS Appl. Mater. Interfaces.

[cit9] Guo C. F., Ren Z. (2015). Mater. Today.

[cit10] Azani M. R., Hassanpour A., Carcelén V., Gibaja C., Ballesté R. M., Zamora F., Granados D. (2016). Applied Materials Today.

[cit11] Langley D., Giusti G., Mayousse C., Celle C., Bellet D., Simonato J. P. (2013). Nanotechnology.

[cit12] Li B., Ye S., Stewart I. E., Alvarez S., Wiley B. J. (2015). Nano Lett..

[cit13] Andrés L. J., Menéndez M. F., Gómez D., Martínez A. L., Bristow N., l Kettle J., Menéndez A., Ruiz B. (2015). Nanotechnology.

[cit14] Abbasi N. M., Yu H., Wang L., Abdin Z. U., Amer W. A., Akram M., Khalid H., Chen Y., Saleem M., Sun R., Shan J. (2015). Mater. Chem. Phys..

[cit15] Lee E. J., Chang M. H., Kim Y. S., Kim J. Y. (2013). APL Mater..

[cit16] Mayousse C., Celle C., Moreau E., Mainguet J. F., Carella A., Simonato J. P. (2013). Nanotechnology.

[cit17] Gebeyehu M. B., Chala T. F., Chang S. Y., Wu C. M., Lee J. Y. (2017). RSC Adv..

[cit18] Pradel K. C., Sohn K., Huang J. (2011). Angew. Chem., Int. Ed..

[cit19] Azani M. R., Hassanpour A. (2019). ChemistrySelect.

[cit20] Zhang P., Wei Y., Ou M., Huang Z., Lin S., Tu Y., Hu J. (2018). Mater. Lett..

[cit21] Rui Y., Zhao W., Zhu D., Wang H., Song G., Swihart M. T., Wan N., Gu D., Tang X., Yang Y., Zhang T. (2018). Nanomaterials.

[cit22] Chang M., Cho H., Kim Y. S., Lee E. J., Kim J. Y. (2014). Nanoscale Res. Lett..

[cit23] Ran Y., He W., Wang K., Ji S., Ye C. (2014). Chem. Commun..

[cit24] Coskun S., Aksoy B., Unalan H. E. (2011). Cryst. Growth Des..

